# Characterization of mRNA Signature in Milk Small Extracellular Vesicles from Cattle Infected with Bovine Leukemia Virus

**DOI:** 10.3390/pathogens12101239

**Published:** 2023-10-13

**Authors:** Md. Matiur Rahman, Hinata Ishikawa, Marika Yamauchi, Shigeo Takashima, Yuji O. Kamatari, Kaori Shimizu, Ayaka Okada, Yasuo Inoshima

**Affiliations:** 1 Laboratory of Food and Environmental Hygiene, Cooperative Department of Veterinary Medicine, Gifu University, Gifu 501-1112, Japan; 2 Department of Medicine, Faculty of Veterinary, Animal and Biomedical Sciences, Sylhet Agricultural University, Sylhet 3100, Bangladesh; 3 Division of Genomics Research, Life Science Research Center, Gifu University, Gifu 501-1112, Japan; 4 Institute for Glyco-Core Research (iGCORE), Gifu University, Gifu 501-1112, Japan; 5 The United Graduate School of Drug Discovery and Medical Information Sciences, Gifu University, Gifu 501-1112, Japan; 6 Division of Instrumental Analysis, Life Science Research Center, Gifu University, Gifu 501-1112, Japan; 7 Education and Research Center for Food Animal Health, Gifu University (GeFAH), Gifu 501-1112, Japan; 8 Joint Graduate School of Veterinary Sciences, Gifu University, Gifu 501-1112, Japan

**Keywords:** bovine milk small extracellular vesicles, low proviral load, high proviral load, bovine leukemia virus transmission, mRNA signature

## Abstract

This study aimed to characterize the mRNA signature of milk small extracellular vesicles (sEVs) from BLV-infected cattle. A total of 23 mRNAs, which showed greater abundance in milk sEVs from BLV-infected cattle compared to those from BLV-uninfected (control) cattle, were identified through microarray analyses conducted in our previous study. To assess the significance of these differences in mRNA abundance, milk was collected from six control cattle and twenty-six cattle infected with BLV. The infected cattle were categorized into two distinct groups based on their proviral loads: a group of eight cattle with low proviral loads (LPVL), characterized by <10,000 copies per 10^5^ white blood cells (WBC), and a group of eighteen cattle with high proviral loads (HPVL), marked by ≥10,000 copies per 10^5^ WBC. The qPCR analysis quantified 7 out of 23 mRNAs, including *BoLA*, *CALB1*, *IL33*, *ITGB2*, *MYOF*, *TGFBR1*, and *TMEM156*, in the milk sEVs from control cattle, LPVL cattle, and HPVL cattle. Significantly, the average relative expression of *CALB1* mRNA in milk sEVs was higher in LPVL cattle compared to HPVL cattle and control cattle (*p* < 0.05), while it was relatively lower in HPVL cattle compared to LPVL cattle and control cattle (*p* > 0.05). Likewise, the average relative expression of *TMEM156* mRNA in milk sEVs was significantly higher in LPVL cattle compared to HPVL cattle (*p* < 0.05), and relatively lower in HPVL cattle compared to LPVL cattle and control cattle (*p* > 0.05). The results indicate distinct patterns of *CALB1* and *TMEM156* mRNA levels in milk sEVs, with higher levels observed in LPVL cattle and lower levels in HPVL cattle. The current study could provide essential information to comprehend the complexities during the progression of BLV infection and direct the exploration of mRNA biomarkers for monitoring the clinical stage of BLV infection.

## 1. Introduction

The bovine leukemia virus (BLV) is a retrovirus that causes the most commonly reported neoplastic disease, called enzootic bovine leukosis (EBL), in cattle (*Bos taurus* and *Bos indicus*) and is characterized by B-cell lymphosarcoma [1]. BLV is transmitted either horizontally or vertically through the transfer of biological fluid contaminated with lymphocytes containing the BLV provirus integrated into the host genome DNA, such as blood and saliva [1,2]. Previous studies have also reported that BLV infection is transmitted through the biting of blood-sucking flies such as those in the *Tabanidae* family [3] and is related to practices such as breeding by natural service [4] or artificial insemination [5]. The World Organization for Animal Health has listed EBL as a worldwide distributed disease that has a significant impact on international trade [6] due to the restriction of the export–import of meat and milk from infected animals. BLV-infected cattle cause direct and indirect losses to the farmer, including increased replacement costs, loss of income from condemned carcasses of culled cattle, increased infertility, decreased milk production, less body gain, susceptibility to other diseases, and the inability to export cattle semen and embryos to countries that maintain BLV control programs [7]. Importantly, there is no availability of a BLV vaccine and no treatment against BLV infection [8].

Although BLV infection is found worldwide, some countries have successfully eradicated BLV infection, including Belgium, Denmark, Germany, Finland, the Netherlands, and the United Kingdom, by implementing nationwide surveys and the slaughtering of BLV-infected cattle [9]. However, the prevalence of BLV infection is high in many countries, including Japan and the USA [10]. In Japan, a nationwide serological survey reported that the presence of antibodies against BLV was 40.9% and 28.7% in dairy and beef breeding cattle, respectively [11]. Although the prevalence rate of BLV is high, only 1–5% of BLV-infected cattle develop EBL [12]. Among the BLV-infected cattle, approximately 70% of BLV-infected cattle do not show any lifelong clinical signs, and 30% of them develop persistent lymphocytosis. A previous study reported that the blood of BLV-infected cattle with persistent lymphocytosis contained high viral copy numbers and has been used as an indicator of disease progression in BLV-infected cattle [13]. Another study reported that host biological factors may feature an important function in the stages of BLV infection from early infection to the development of persistent lymphocytosis to lymphoma [14]. Taken together, these findings indicate that changes in the expression patterns of the host biological molecules are crucial for virological analysis or monitoring of the stages of BLV infection.

Extracellular vesicles (EVs) are membranous vesicles that can be found in most bodily fluids, including blood, milk, urine, saliva, ascites fluid, amniotic fluid, body sweat, and tears [15,16]. According to the size, biogenesis, and release pathways, EVs have different terms such as exosomes, ectosomes, shedding vesicles, and micro vesicles [17]. EVs with a size of ~200 nm are referred to as “exosomes” [18]. According to the Minimal Information for Studies of Extracellular Vesicles 2018 (MISEV2018) guidelines by the International Society for Extracellular Vesicles, the use of the term “small EVs” (sEVs) rather than “exosomes” is recommended [17]. Bovine milk also contains sEVs encapsulating mRNAs, miRNAs, DNAs, proteins, and lipids which are biologically important for many aspects, including intercellular communication [19,20]. For example, a previous study reported that bovine milk sEVs contain *Transforming Growth Factor beta* (*TGF-β*) mRNA that plays a role in the development of intestinal barrier function in human infants, initiation of IgA production, and mucosal immunity during infancy [21]. Moreover, another study reported that miRNAs such as bta-miR-1246 and hsa-miR-424-5p in bovine milk sEVs provide novel information on the pathological condition of the EBL in cattle [22].

Our previous studies reported that BLV infection may cause profound effects on host molecules, resulting in changes in encapsulated biological materials such as mRNAs and proteins in bovine milk sEVs [23,24]. The results indicated that the encapsulated biological materials, such as mRNAs derived from the host in bovine milk sEVs, should be useful in investigating the intracellular expression profiles during BLV infection. As a result, we hypothesized that the mRNA signature in milk sEVs from BLV-infected cattle with a low proviral load (LPVL) to high proviral load (HPVL) (early infection to late stage of infection) could be benchmark research for the exploration of immunological dynamics in the host; however, it has not yet been established. Therefore, the purpose of the current study is to characterize the mRNA signature of milk sEVs from BLV-infected cattle for the monitoring of the infection stage of LPVL to HPVL. In this study, *CALB1* and *TMEM156* mRNAs were found to be higher in milk sEVs from LPVL cattle (*p* < 0.05) and decreased in milk sEVs from HPVL cattle.

## 2. Materials and Methods

### 2.1. Clinical Status of the Cattle

Blood samples of 10 mL were taken from a total of 106 Holstein-Friesian dairy cattle from different farms, with ages ranging from 19 to 133 months. The blood was directly allocated to vacuum blood collection tubes with or without heparin (VENOJECT II, VP-H070K or VP-AS076K, Terumo, Tokyo, Japan). WBC and lymphocyte counts were determined using the automatic cell counter Celltac α (Nihon Kohden, Tokyo, Japan). After counting the WBCs and lymphocytes, 1.3 mL of each anticoagulated blood sample was centrifuged at 2500× *g* at 25 °C for 15 min for plasma separation by using a centrifuge MX-307 (Tomy Seiko, Tokyo, Japan). The supernatant plasma was transferred into a 1.3 mL tube separately from the bottom blood pellet for further use.

#### 2.1.1. Identification of Serum Antibodies against BLV

Serum was separated from blood without heparin by centrifugation at 3000× *g* for 15 min at 25 °C by using a centrifuge MX-307. Anti-BLV antibody levels in serum were determined by using an anti-BLV antibody ELISA kit (JNC, Tokyo, Japan) according to the manufacturer’s instructions.

#### 2.1.2. Identification of the BLV Provirus

To isolate WBCs from blood, 1 mL of 0.83% ammonium chloride with 0.01% EDTA solution was added into 300 µL of blood pellet, mixed by inverting several times, followed by incubation at 25 °C for 5 min, centrifugation at 2500× *rpm* at 25 °C for 10 min, and washing twice with 1 mL of phosphate buffered saline (PBS). DNA was isolated from WBCs using the DNeasy Blood and Tissue Kit (69506, Qiagen, Hilden, Germany) according to the manufacturer’s instructions, and the DNA concentration was measured using a spectrophotometer NanoDrop Lite (Thermo Fisher Scientific, Waltham, MA, USA). Nested PCR was carried out with primers for the envelope or pX region of BLV according to the protocols as described previously [25,26] using a thermal cycler Veriti 200 (Applied Biosystems, Waltham, MA, USA) in a total reaction mixture of 20 µL containing 0.5 units of polymerase from GoTaq Hot Start Green Master Mix (M5122, Promega, Madison, WI, USA) or SapphireAmp Fast PCR Master Mix (RR350A, Takara Bio, Kusatsu, Japan), 1 µL of each of forward and reverse primers, 1 µL of extracted WBC DNA (100 to 400 ng), and PCR grade water. The thermal cycling conditions were as follows: 95 °C for 2 min, followed by 35 cycles of 94 °C for 45 sec, 62 °C for 30 sec, 72 °C for 30 sec, and finally 72 °C for 4 min. The PCR products were electrophoresed on a 1% agarose gel and were visualized by ethidium bromide staining.

#### 2.1.3. Assessment of BLV Proviral Load (PVL)

A previous study reported that BLV-infected cattle with HPVL in their blood were considered as cattle at high risk for BLV transmission which might be one of the factors of disease progression [27]. As a result, the current study emphasized the measurement of PVL from BLV-infected cattle by using qPCR analysis. The amplification was performed in a reaction mixture containing 10 µL of THUNDERBIRD Probe qPCR Mix (A4250K, Toyobo, Osaka, Japan), 0.3 µL of CoCoMo-BLV Primer/Probe (A803, Riken Genesis, Tokyo, Japan), 5 µL of WBC DNA, and PCR grade water. For the PVL quantification, BLV BoLA-DRA gene Plasmid DNA (A804, Riken Genesis) was used from the kit, and BLV proviral DNA was determined using a Thermal Cycler Dice Real Time System III (TP970, Takara Bio) as described previously [23,24].

#### 2.1.4. Measurement of the Total Lactate Dehydrogenase (LDH) Activity and LDH Isozymes Ratio

A previous study indicated that the total LDH isozyme activity in blood reflected the disease progression of EBL; in particular, increased percentages of the LDH 2+3 isozyme ratio were established as a key parameter for the diagnosis of lymphosarcoma [28]. Therefore, the current study also focused on the total LDH activity and LDH isozyme ratio in blood from BLV-infected cattle with LPVL and HPVL. The total LDH activity (IU/L) was measured using an auto analyzer JCS-BM6050 (JEOL, Tokyo, Japan) using an enzymatic method (L-Type Wako LD IF or L-Type Wako J, Fujifilm Wako Pure Chemical, Osaka, Japan). The percentage of the LDH isozyme ratio was measured by a Hydrasys 2 Scan (Sebia, Paris, France) using HYDRAGEL 7 ISO-LDH (Sebia), and was conducted by a clinical laboratory testing company, Fujifilm Vet Systems (Tokyo, Japan).

### 2.2. Milk Samples

Fresh raw milk was collected from 32 Holstein-Frisian cattle from different farms located in Gifu Prefecture, Japan. All hygienic parameters were observed both before and after milk collection from the cattle. Milk was obtained from individual cattle using hand milking. Collected milk samples were promptly shipped to our laboratory and maintained at 4 °C until further processing. The clinical status of these cattle is shown in Table 1.

#### 2.2.1. Isolation and Characterization of Milk sEVs

Milk sEVs were isolated and purified as described previously with slight modifications [23,24,29,30,31]. Casein was removed from the milk using an acetic acid treatment followed by filtration of the supernatant (whey) by using 1.0-, 0.45-, and 0.2- µm pore-size filters (GA-100, C045A047A, and C020A047A, Advantec, Tokyo, Japan). Approximately 50 mL of filtrated whey was pelleted by ultracentrifugation (UC) at 100,000× *g* at 4 °C for 1 h in a P42A angle rotor (Hitachi Koki, Hitachinaka, Japan) using a Himac CP60E ultracentrifuge (Hitachi Koki). After the UC, the supernatant was decanted, and the pellet was collected from the centrifuge tube into a 13PET tube (Hitachi Koki) and resuspended in 10 mL of PBS. Then, UC was performed at 100,000× *g* at 4 °C for 1 h in a P42ST swing rotor (Hitachi Koki). The supernatant was decanted again and the pelleted milk sEVs were collected and stored at 4 °C or −80 °C for further application. 

According to the MISEV2018 guidelines [17], milk sEVs were characterized by using transmission electron microscopy (TEM), nanoparticle tracking analysis (NTA), and Western blotting (WB) as described previously [23,24,29,30,31], with slight modifications. For TEM observation, the milk sEV pellet solution was diluted ten times using distilled water and taken onto glow-discharged carbon support films on copper grids. After that, the milk sEVs were stained by using 2% uranyl acetate. The stained copper grid was placed in a silicon chamber for drying. The morphology of the milk sEVs was observed under a JEM-2100F (JEOL, Tokyo, Japan) electron microscope at 200 kV. Further, the NTA of milk sEVs was performed using a NanoSight LM10V-HS, NTA 3.4 instrument (Malvern Panalytical, Malvern, UK) by an assigning company (Quantum Design Japan, Tokyo, Japan). In the WB analysis, the proteins transferred to polyvinylidene difluoride (PVDF) blotting membranes were blocked with 5% skimmed milk in Tris-buffered saline (0.1M Tris-HCl (pH 8.0) and 0.03M NaCl) containing 0.1% Tween-20 (TBST) at 25 °C for 20 min. Then, the primary antibodies for the detection of the sEVs, surface-marker protein CD63 (1:200, M-13, SC-31214, Santa Cruz Biotechnology, Santa Cruz, CA, USA), internal-marker protein HSP70 (1:100, N27F3-4, ADI-SPA-820, Enzo Life Science, Farmingdale, NY, USA), and contaminant-marker protein apoA1 (1:100, B-10, SC-376818, Santa Cruz Biotechnology), were added to the PVDF membranes and incubated at 25 °C for 1 h. The secondary antibodies anti-goat IgG donkey antibody (1:2000, SC-3851, Santa Cruz Biotechnology) or anti-mouse IgG antibody (1:2000, 7076, Cell Signaling Technologies, Danvers, MA, USA) conjugated with horseradish peroxidase were incubated with the PVDF membrane at 25 °C for 1 h. Finally, the Pierce ECL Plus substrate (Thermo Fisher Scientific, Waltham, MA, USA) was added to the membrane to detect the labeled antibodies, and the protein images were visualized by using the chemiluminescence imager ChemiDoc XRS+ (Bio-Rad Laboratories, Hercules, CA, USA).

#### 2.2.2. Extraction of RNA and cDNA Synthesis

The total RNA was obtained from milk sEVs using a Maxwell RSC simply RNA Tissue Kit (AS1340, Promega) according to the manufacturer’s instructions. The concentration of RNA was measured by NanoDrop Lite (Thermo Fisher Scientific). After the total RNA extraction from milk sEVs, extraneous DNA was removed by treatment with DNase I (18068-015, Invitrogen, Carlsbad, CA, USA). After that, complementary DNA (cDNA) was prepared from 150 ng RNA using a PrimeScript RT Master Mix (RR036A, Takara Bio) following the manufacturer’s guidelines.

#### 2.2.3. qPCR Analysis

A total of 23 mRNAs in milk sEVs from BLV-infected cattle as compared to control cattle were selected from the microarray data of our previous study [23] based on their fold change value, their roles on cellular and metabolic activity, and their involvement in cancer or tumor progression and invasion (Supplementary Table S1) [32,33,34,35,36,37,38,39,40,41,42,43,44,45,46,47,48,49,50,51,52,53,54]. All 23 primers for qPCR analysis were designed by using Primer BLAST software from the National Center for Biotechnology Information (http://www.ncbi.nlm.nih.gov/tools/primer-blast/, accessed on 11 November 2020) considering the appropriate amplicon sizes (100–250 bp) (Supplementary Table S2). A total of 20 µL of reaction volume was prepared that included 10 µL of PowerUp SYBR Green Master Mix (Thermo Fisher Scientific), 1 µL each of forward and reverse primers (0.5 μM), 2 µL of cDNA (15 ng/µL), and 6 µL of nuclease-free water. After that, qPCR was conducted by utilizing a StepOnePlus thermal cycler (Applied Biosystems) in a 96-well optical plate (Applied Biosystems). The following amplification conditions were maintained: 50 °C for 2 min, 95 °C for 2 min, 40 cycles: 95 °C for 3 sec (denaturation) and 60 °C for 30 sec (annealing and extension). Notably, qPCR was conducted in duplicate for each mRNA (technical replicates). A dissociation procedure using temperatures of 95 °C for 15 sec, 60 °C for 1 min, and 95 °C for 15 sec was used and a melt curve analysis was performed to validate the specificity of the reactions and the presence of primer dimers. The mRNA amounts relative to the controls (mean of controls = 1) were determined using the ΔΔCt method [55].

### 2.3. Statistical Analysis

Quantitative data of the relative amount of mRNAs in milk sEVs among three groups such as control cattle (*n* = 6), LPVL cattle (<10,000 copies/10^5^ WBC) (*n* = 8), and HPVL cattle (≥10,000 copies/10^5^ WBC) (*n* = 18) were compared using a one-way ANOVA according to the previous study [56]. Data were expressed as the means ± standard deviation and analyzed for statistical significance by the Kruskal–Wallis *H*-test (*p* < 0.05). To evaluate the predictive value of mRNAs, a receiver operating characteristic (ROC) curve analysis was applied by using EZR software ver. 1.54 (http://www.jichi.ac.jp/saitama-sct/SaitamaHP.files/statmedEN.html, accessed on 14 September 2021) [57] and the area under the curve (AUC) was compared. In addition, the correlation coefficient between the mRNAs in milk sEVs and hematological parameters from BLV-infected cattle was evaluated.

## 3. Results

### 3.1. Clinical Status of the Cattle

The clinical status of the BLV-infected cattle and their hematological and serum chemical profiles were investigated and are shown in Table 1. For the confirmation of BLV infection in cattle, the BLV provirus and BLV antibody were tested using nested PCR and ELISA. The result showed that BLV-infected cattle nos. 7–14 had a LPVL (<10,000 copies/10^5^ WBC) and nos. 15–32 had an HPVL (≥10,000 copies/10^5^ WBC), indicating the clinical stage of BLV progression from early stage to late stage. The WBC and lymphocyte counts were over 9000/µL and over 5000 /µL indicating “suspect” according to the key EC parameter [58].

### 3.2. Isolation and Characterization of Bovine Milk sEVs

**Figure 1:**
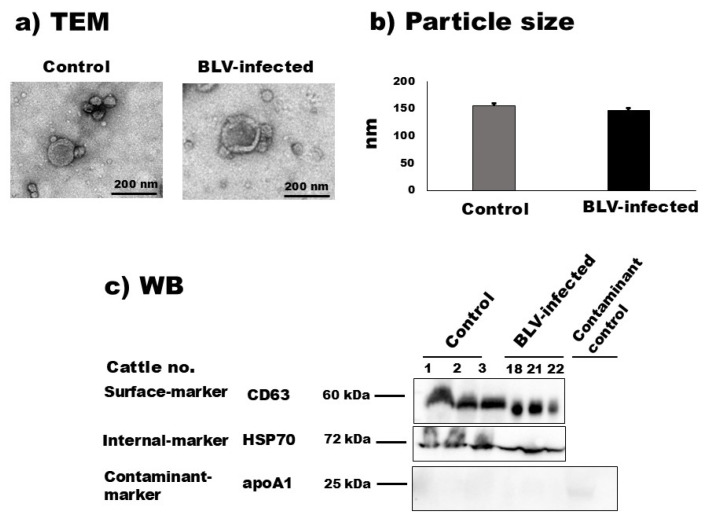
Characterization of milk small extracellular vesicles (sEVs). (**a**) Morphological features of bovine milk sEVs from control cattle and bovine leukemia virus (BLV)-infected cattle were observed by transmission electron microscopy analysis (scale bar indicates 200 nm). (**b**) Nanoparticle tracking analysis determined the size distribution of milk sEVs from control and BLV-infected cattle (average mean peak size below 200 nm in diameter), and there was no significant difference observed between them (*p* > 0.05). (**c**) Bovine milk sEVs surface-, internal-, and contaminant-marker proteins, CD63, HSP70, and apoA1, were detected by Western blot analysis, indicating that bovine milk sEVs were successfully isolated according to the minimal information for studies of extracellular vesicles 2018 guidelines [17]. BLV, bovine leukemia virus; TEM, transmission electron microscopy; and WB, Western blot.

According to the TEM observation, the morphological characteristics of bovine milk sEVs from uninfected cattle and BLV-infected cattle are demonstrated in Figure 1 and Supplementary Figure S1. The results showed a homologous circular bilayer structure (Figure 1a). The NTA demonstrated that the average peak mode (intensities) for the particle size distribution of milk sEVs from uninfected cattle and BLV-infected cattle were 156.0 nm ± 10.72 nm and 147.66 nm ± 8.29 nm (Figure 1b) (*p* > 0.05), respectively. WB analysis successfully identified milk sEV surface-marker protein CD63, internal-marker protein HSP70, and contaminant-marker protein apoA1 by using anti-CD63, –HSP70, and -apoA1 antibodies (Figure 1c) and revealed less contaminant protein apoA1 in milk sEVs compared with the contaminant control sample (unfiltered whey). Taken together, these results indicated the presence and successful isolation of milk sEVs based on the MISEV2018 guidelines [17].

### 3.3. qPCR Analysis

**Figure 2:**
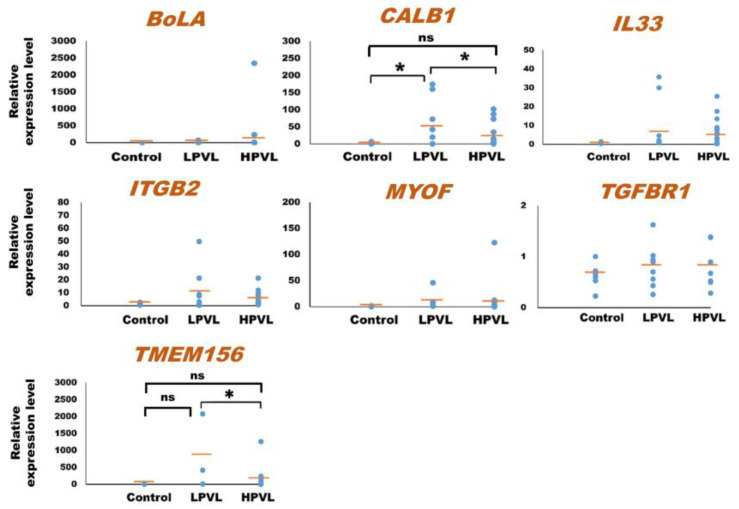
Quantification of mRNA in milk small extracellular vesicles (sEVs) by a quantitative real-time polymerase chain reaction. The dot plots compare the relative amount of mRNA in milk sEVs from bovine leukemia virus-infected cattle with 8 low proviral loads (LPVL) and 18 high proviral loads (HPVL) compared with 6 control cattle. Each data point in the plot represents an individual cattle (*n* = 32). In a one-way ANOVA, the statistical significance was calculated by the Kruskal–Wallis *H*-test (* *p* < 0.05) and the bar indicates the average of the relative amount of mRNA in milk sEVs from LPVL cattle and HPVL cattle compared with control cattle. sEVs, small extracellular vesicles; ns, not significant; BLV, bovine leukemia virus; LPVL, low proviral load (<10,000 copies/10^5^ WBCs DNA); and HPVL, high proviral load (≥10,000 copies/10^5^ WBCs DNA).

As for the 23 mRNAs, preliminary quantification by qPCR analysis was carried out using one control cattle (no. 1) and one BLV-infected cattle (no. 18) to determine whether these mRNAs were detected or not. In a prior investigation, ACTB was used as an internal control mRNA in milk sEVs for the normalization in qPCR [31,59]. Among the 23 mRNAs, a total of 7 mRNAs including *bovine major histocompatibility complex* (*BoLA*), *calbindin 1* (*CALB1*), *interleukin 33* (*IL33*), *integrin subunit beta 2* (*ITGB2*), *myoferlin* (*MYOF*), *transforming growth factor beta receptor 1* (*TGFBR1*), and *transmembrane protein 156* (*TMEM156*) were detected by qPCR using the primers designed in this study. Finally, the relative amount of these mRNAs in milk sEVs was checked by qPCR analysis using 32 control (*n* = 6) and BLV-infected (*n* = 26) cattle. Importantly, the BLV-infected cattle were divided into two groups according to the criteria of PVL as LPVL, <10,000 copies/10^5^ WBC (*n* = 8), and HPVL, ≥10,000 copies/10^5^ WBC (*n* = 18) (Table 1) [33]. The results showed that the average relative amounts of *CALB1*, *IL33*, *ITGB2*, *MYOF*, and *TMEM156* mRNAs were lower in milk sEVs from HPVL cattle compared to LPVL cattle but maintained at slightly higher levels compared to control cattle (Figure 2). Importantly, in the case of *CALB1* mRNA, there was a significant difference observed in the milk sEVs from LPVL cattle compared with that of control cattle (*p* < 0.05). Notably, the average relative amount of *BoLA* and *TGFBR1* in milk sEVs from LPVL cattle and control cattle was almost the same. On the other hand, the average relative amount of *CALB1*, *IL33*, *ITGB2*, *MYOF*, and *TMEM156* mRNAs were lower in milk sEVs from HPVL cattle compared with LPVL cattle but maintained little bit high amount compared with control cattle. However, in the case of *CALB1* and *TMEM156* mRNAs, a significant difference was observed in the milk sEVs from LPVL cattle vs. HPVL cattle (*p* < 0.05). The average relative amount of *BoLA* mRNA in milk sEVs from HPVL cattle was high compared with LPVL cattle and control cattle but not significantly different (*p* > 0.05). In the case of *TGFBR1* mRNA, the average relative amount in the milk sEVs from HPVL cattle was the same with LPVL cattle and control cattle. Notably, the average relative amount of *CALB1* and *TMEM156* in the milk sEVs from HPVL cattle compared with control cattle did not differ significantly (*p* > 0.05). The relative expression level of *CALB1* and *TMEM156* mRNAs in accordance with the control, LPVL, and HPVL cattle were demonstrated as a plot diagram in Supplementary Figure S2.

**Figure 3:**
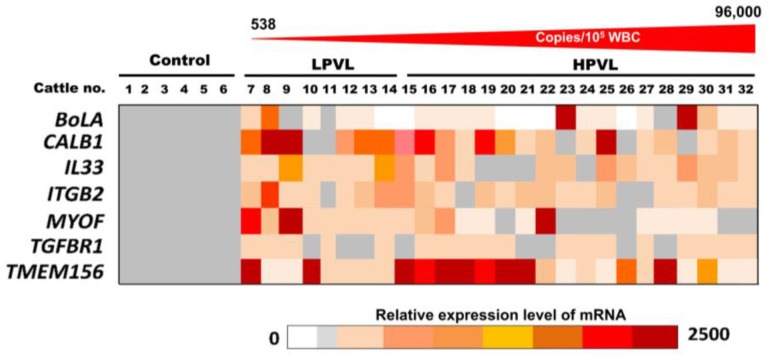
The relative amount of mRNAs in milk small extracellular vesicles (sEVs) by quantitative real-time polymerase chain reaction. The color block indicates the relative amount of mRNA in milk sEVs from bovine leukemia virus-infected cattle with 8 low proviral loads and 18 high proviral loads. Each data point in the plot represents an individual cattle (*n* = 26). sEVs, small extracellular vesicles; LPVL, low proviral load (<10,000 copies/10^5^ white blood cells (WBCs) DNA); and HPVL, high proviral load (≥10,000 copies/10^5^ WBCs DNA).

The relative amount of the seven mRNAs, *BoLA*, *CALB1*, *IL33*, *ITGB2*, *MYOF*, *TGFBR1*, and *TMEM156* in the milk sEVs from 6 control cattle and 26 BLV-infected cattle (8 LPVL and 18 HPVL) are displayed as a color block in Figure 3. The various colors indicate the different relative amounts of the individual mRNAs in the milk sEVs among the 32 cattle. The relative amount of *CALB1* mRNA was very high in LPVL cattle (cattle nos. 8 and 9) and HPVL cattle (cattle nos. 16, 19, and 25) compared with control cattle. On the other hand, the relative amount of *TMEM156* mRNA was also very high in LPVL cattle (cattle nos. 7 and 10) and HPVL cattle (cattle nos. 15–21, and 28) compared with control cattle. The relative amounts of other mRNAs, *IL3*3, *ITGB2*, *MYOF*, and *TGFBR1* in milk sEVs from LPVL cattle and HPVL cattle did not fluctuate as much compared with the control cattle. Notably, the relative amount of *BolA* mRNA in milk sEVs from LPVL cattle was not higher compared with the control but only two HPVL cattle (cattle nos. 23 and 29) showed a very high amount compared with LPVL and control cattle. Additionally, a few of the LPVL cattle (cattle no. 13 and 14) and HPVL cattle (cattle nos. 15, 20, 21, 22, and 26) did not show any detectable amount of *BoLA* mRNA in milk sEVs. Taken together, the results indicate that the relative amounts of *CALB1* and *TMEM156* mRNAs in milk sEVs from LPVL cattle and HPVL cattle were comparable to those of the control cattle.

**Figure 4:**
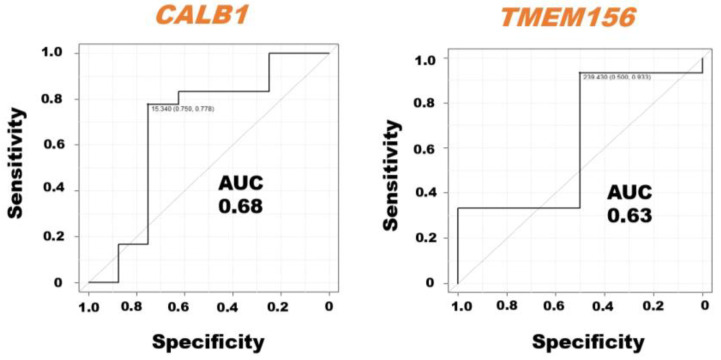
Receiver operating characteristic (ROC) curve analysis of the mRNAs in milk small extracellular vesicles (sEVs). The ROC curve analysis of mRNAs in milk sEVs from low proviral load (LPVL) cattle and high proviral load (HPVL) cattle. *CALB1* and *TMEM156* showed an AUC of 0.68 and 0.63, respectively, in the milk sEVs from bovine leukemia virus-infected cattle with LPVL and HPVL. AUC, area under the curve; ROC, receiver operating characteristic; LPVL, low proviral load (<10,000 copies/10^5^ white blood cells (WBCs) DNA); HPVL, high proviral load (≥10,000 copies/10^5^ WBCs DNA); and sEVs, small extracellular vesicles.

The ROC curve analysis demonstrated the AUC with sensitivity and specificity of the *CALB1* and *TMEM156* mRNAs in milk sEVs from LPVL cattle and HPVL cattle (Figure 4). We excluded the *BoLA*, *IL33*, *ITGB2*, *MYOF*, and *TGFBR1* mRNAs from the ROC curve analysis because the average relative amount of these mRNAs in the milk sEVs from LPVL cattle and HPVL cattle did not show a significant difference in qPCR analysis. The ROC curve analysis revealed the high AUC value of *CALB1,* i.e., 0.68, in the milk sEVs from LPVL cattle and HPVL cattle (95% CI: 0.2646–0.9606). On the other hand, the AUC of *TMEM156* in the milk sEVs from LPVL cattle and HPVL cattle was also high, i.e., 0.63 (95% CI: 0.4005–1.0). The sensitivity and specificity of the *CALB1* mRNAs in the milk sEVs were 75% and 77.8%, respectively. On the other hand, the sensitivity and specificity of the *TMEM156* mRNAs in the milk sEVs were 50% and 93.3%, respectively.

Additionally, the correlation coefficient result is demonstrated in Table 2. The results showed that *BoLA* mRNA was positively but less strongly correlated with the PVL of the BLV-infected cattle. On the other hand, *CALB1*, *IL33*, *ITGB2*, *MYOF*, *TGFBR1,* and *TMEM156* mRNAs were negatively but not strongly correlated with the PVL of the BLV-infected cattle. The correlation coefficient results also showed that no such strong correlation was found between the mRNAs in milk sEVs with other hematological parameters such as age, WBC count, lymphocyte count, and LDH isozymes 2+3% from BLV-infected cattle (Table 2). 

## 4. Discussion

In this study, we attempted to characterize the mRNA signature of milk sEVs from BLV-infected cattle with LPVL and HPVL compared with control cattle. The study found that the levels of two mRNAs, *CALB1* and *TMEM156,* were higher in milk sEVs from LPVL cattle compared to those in control cattle (*p* < 0.05).

In the present study, *CALB1* mRNA was significantly more abundant in milk sEVs from LPVL cattle compared with control cattle (*p* < 0.05). However, the amount of *CALB1* mRNA was decreased in HPVL cattle and significantly differed in the milk sEVs from LPVL cattle compared with HPVL cattle (*p* < 0.05). *CALB1* mRNA is a member of the calcium-binding protein family that is normally expressed in osteoblasts and is involved in the formation of a mineralized bone matrix by maintaining low levels of intracellular calcium [50]. A previous study reported that the calcium levels in blood from BLV-infected cattle were considerably lower than uninfected cattle and stated that variation in the calcium level could be attributable to different phases of leukosis [60]. This finding is consistent with our present study findings, where the *CALB1* mRNA in milk sEVs from HPVL cattle was significantly low compared with LPVL cattle and control cattle, indicating that the relative amount of *CALB1* mRNA may decrease during the progression of BLV infection. However, few previous studies reported that a higher amount of *CALB1* mRNA promoted the proliferation and colony formation of ovarian cancer cells by inhibiting senescence [61,62]. Our findings indicated that the relatively higher amount of *CALB1* mRNA in milk sEVs was significantly associated with forthcoming LPVL cattle and the lower amount of *CALB1* mRNA in milk sEVs was significantly associated with HPVL cattle.

A recent study reported that the relative amount of *TMEM156* mRNA was significantly higher in milk sEVs from EBL cattle compared with control cattle, indicating that *TMEM156* is a potential biomarker for the prediction of EBL in cattle [59]. In the current study, the amount of *TMEM156* mRNA was higher and significantly different in the milk sEVs from LPVL cattle and HPVL cattle (*p* < 0.05). However, the *TMEM156* mRNA did not show any significant difference in the milk sEVs from LPVL cattle vs. control cattle and HPVL cattle vs. control cattle. In the course of increasing PVL in BLV-infected cattle, the amount of *TMEM156* mRNA may decrease in milk sEVs from HPVL cattle compared with LPVL cattle. A previous study also reported that the relative amount of *TMEM156* mRNA was higher in various cancer cell lines such as prostate, breast, and liver cancers [33]. However, the function of *TMEM156* mRNA in cancer has been poorly explored. BLV is an oncogenic virus, which is one possible explanation for why the relatively lower amount of *TMEM156* mRNA may be associated with a worse outcome in HPVL cattle. It is possible that the relative amount of *TMEM156* mRNA decreases in the milk sEVs from HPVL cattle before the onset of EBL. Nevertheless, despite the importance of *TMEM156* mRNA in carcinogenesis, its biological significance is still poorly understood and limited.

In this study, the average relative amount of *BoLA* mRNA in milk sEVs was higher in LPVL cattle and HPVL cattle compared with control cattle. Although the amount of *BoLA* mRNA in the milk sEVs was not significantly different, the role of *BoLA* mRNA in BLV infection is quite important. The average amount of *BoLA* mRNA in milk sEVs was high; however, the amount of *BoLA* mRNA in milk sEVs from a few HPVL cattle was not detected by qPCR analysis. Moreover, most of the HPVL cattle showed a low relative amount of *BoLA* mRNA in their milk sEVs with qPCR. As a result, we suggest that in the clinical stage of BLV infection, an increase in PVL may be defended by a host differential immune response, triggering different subsequent immune responses and resulting in the inconsistency in the amount of *BoLA* mRNA in the milk sEVs from HPVL cattle. A previous study reported that *BoLA* mRNA is functionally important and highly polymorphic in nature, playing an important function in antigen presentation and immune responsiveness, and, thus, linked to infectious diseases, including BLV [63,64]. *BoLA-DRB3* polymorphism is highly related to susceptibility to lymphoma or resistance and susceptibility to PVL in cattle [65]. These findings are consistent with our present study results, although we did not check the susceptibility or polymorphism of *BoLA* mRNA in the milk sEVs from BLV-infected cattle.

In addition, we investigated the correlations between the mRNA signature with several factors, including age, WBC count, lymphocyte count, PVL, total LDH, and LDH 2+3% (Table 2). Previous studies have reported that these factors are associated with the progression of EBL in cattle and act as potential biomarkers [28,66]. However, the present study did not identify any strong correlation between these parameters with the number of mRNAs in milk sEVs, according to the BLV infection stage. The study result is partially consistent with the findings by Hiraoka et al. [59] in that factors such as age, WBC count, lymphocyte count, and the PVL exception of LDH isozymes 2+3% showed no strong correlation with mRNA biomarker candidates in EBL. Although the current study focused on LPVL cattle and HPVL cattle, an association with various factors should be considered for processing the clinical stage of the BLV infection. 

However, the current study has a few limitations. First, to validate the mRNAs in milk sEVs, we used a small number of samples during the qPCR analysis. Second, we did not accurately compare our study findings with those of previous studies due to a lack of information, making it difficult to discuss. To overcome these limitations, further experiments could be performed using a large number of samples during qPCR analysis.

## 5. Conclusions

In conclusion, the identified *CALB1* and *TMEM156* mRNA levels were higher in the milk sEVs from LPVL cattle and relatively low in HPVL cattle. As a result, a higher amount of *CALB1* and *TMEM156* mRNAs in milk sEVs could represent LPVL cattle, and a lower amount of *CALB1* and *TMEM156* mRNAs in milk sEVs could represent HPVL cattle. The current study also indicated a baseline for the distinctive pattern of mRNAs in milk sEVs during BLV infection. Further research is needed to determine how these differences occur and how they vary in milk sEVs during the progression of BLV infection from early infection to the onset of EBL in cattle. The findings may allow us to use these mRNAs for a better understanding and monitoring of the clinical stage of BLV infection and the onset of EBL.

## Figures and Tables

**Table 1:** Assessment of BLV infection and the clinical status of cattle used in the validation of the mRNA signature in milk sEVs.

Cattle	Age (Month)	ELISA	Nested PCR	PVL (Copies/10^5^ WBC)	WBC (/µL)	Lymphocyte (/µL)	Key of EC	Total LDH (IU/L)	LDH Isozyme (%)					
									1	2	3	2+3	4	5
Uninfected (Control) Cattle														
1	21	-	-	-	4800	2700	-	881	51.5	26.4	14.9	41.3	4.9	2.3
2	20	-	-	-	9100	4400	-	955	50.0	25.9	15.4	41.3	5.2	3.5
3	33	-	-	-	8600	4200	-	876	52.4	22.6	15.7	38.3	5.2	4.1
4	68	-	-	-	6000	3100	-	839	58.8	25.1	12.4	37.5	2.8	0.9
5	44	-	-	-	5400	2100	-	778	57.5	25.6	12.6	38.2	2.9	1.4
6	116	-	-	-	NT	NT	-	723	63.9	16.6	13.0	29.6	4.3	2.2
BLV-Infected Cattle with LPVL														
7	104	+	+	537.67	10,200	2101	-	691	56.1	21.3	13.0	34.3	4.8	4.8
8	43	+	+	609.43	10,400	4638	-	935	53.1	24.1	14.5	38.6	5.2	3.1
9	112	+	+	2434.07	7000	2282	-	980	45.8	22.1	16.8	38.9	7.0	8.3
10	56	+	+	2794.65	9000	5373	±	966	50.2	23.3	15.0	38.3	7.0	4.5
11	99	+	+	2798.73	5000	2185	-	850	53.8	21.2	14.4	35.6	6.7	3.9
12	41	+	+	5547.39	8500	4259	-	1364	38.8	17.6	19.6	37.2	10.2	13.8
13	82	+	+	6430.49	12,000	3804	-	947	59.5	19.3	16.8	36.1	3.3	1.1
14	52	+	+	9057.53	7400	3656	-	882	55.9	22.0	13.6	35.6	5.0	3.5
BLV-Infected Cattle with HPVL														
15	134	+	+	11,765.44	6400	3296	-	883	52.5	20.4	17.8	38.2	5.9	3.4
16	66	+	+	12,587.92	10,000	4480	-	1001	49.1	24.5	15.5	40.0	6.7	4.2
17	98	+	+	18,984.61	9700	5209	±	848	57.2	21.1	13.0	34.1	5.5	3.2
18	119	+	+	21,205.72	13,600	6623	±	783	51.2	23.0	15.4	38.4	6.7	3.7
19	22	+	+	21,589.79	12,300	6950	-	917	53.3	29.7	13.3	43.0	3.1	0.6
20	55	+	+	27,029.76	18,800	10,171	+	1124	60.7	22.9	9.7	32.6	2.6	4.1
21	85	+	+	29,046.10	14,000	9562	+	915	53.4	20.6	15.9	36.5	5.9	4.2
22	90	+	+	29,877.98	11,500	6739	±	970	53.4	21.4	14.6	36.0	6.3	4.3
23	99	+	+	31,534.06	10,100	5636	±	1025	56.7	20.0	12.6	32.6	6.2	4.5
24	41	+	+	31,802.33	10,600	7378	±	1129	52.5	21.7	15.3	37.0	6.4	4.1
25	65	+	+	42,603.86	19,900	11,303	+	1048	58.9	19.9	11.7	31.6	5.7	3.8
26	139	+	+	46,711.64	9300	4548	-	882	48.3	22.4	15.6	38.0	7.8	5.9
27	52	+	+	47,450.30	11,000	6666	±	1221	60.3	22.2	11.0	33.2	4.4	2.1
28	101	+	+	52,834.18	11,800	6856	±	970	55.3	21.1	13.7	34.8	5.8	4.1
29	124	+	+	60,204.08	10,600	6996	±	961	53.4	22.0	12.9	34.9	7.2	4.5
30	78	+	+	76,860.14	17,100	11,457	+	873	51.8	22.6	16.1	38.7	6.3	3.2
31	53	+	+	87,417.31	24,400	13,713	+	1233	58.1	22.0	13.0	35.0	4.6	2.3
32	76	+	+	95,103.60	21,300	16,273	+	924	51.8	23.4	14.6	38.0	6.5	3.7

+, positive; -, negative; NT, not tested; BLV, bovine leukemia virus; sEVs, small extra cellular vesicles; qPCR, quantitative real-time polymerase chain reaction; ELISA, enzyme-linked immunosorbent assay; PVL, proviral load; WBC, white blood cell; Key of EC, leukosis-key of the European Community (-, normal; ±, suspect, +, lymphocytic) [58]; LPVL, low proviral load; HPVL, high proviral load; and LDH, lactate dehydrogenase. Some of the blood examination data were obtained from our previously published paper [31].

**Table 2:** The correlation coefficient of the mRNAs in milk sEVs with hematological parameters from BLV-infected cattle.

	PVL	Age	WBC	Lymphocyte	Total LDH	LDH 2+3%	*BoLA*	*CALB1*	*MYOF*	*IL33*	*ITGB2*	*TGFBR1*	*TMEM156*
PVL	1.00	0.03	0.73	0.85	0.21	−0.16	0.07	−0.42	−0.06	−0.09	−0.30	−0.04	−0.28
Age	-	1.00	−0.28	−0.28	−0.36	−0.38	0.11	−0.09	0.01	0.36	−0.08	0.08	0.13
WBC	-	-	1.00	0.92	0.25	−0.21	−0.06	−0.09	0.16	−0.27	−0.13	−0.05	−0.26
Lymphocyte	-	-	-	1.00	0.27	−0.13	0	−0.26	0.10	−0.25	−0.19	−0.13	−0.30
Total LDH	-	-	-	-	1.00	−0.11	−0.17	0.03	0.09	−0.04	−0.06	0.01	0.09
LDH 2+3%	-	-	-	-	-	1.00	0.07	0.12	−0.33	−0.07	0.11	−0.25	−0.23
*BoLA*	-	-	-	-	-	-	1.00	−0.11	0.23	−0.01	−0.09	0.02	−0.13
*CALB1*	-	-	-	-	-	-	-	1.00	0.56	0.29	0.62	0.25	0.04
*MYOF*	-	-	-	-	-	-	-	-	1.00	0.17	0.11	0.11	−0.10
*IL33*	-	-	-	-	-	-	-	-	-	1.00	0.26	0.17	.01
*ITGB2*	-	-	-	-	-	-	-	-	-	-	1.00	−0.03	0.02
*TGFBR1*	-	-	-	-	-	-	-	-	-	-	-	1.00	0.04
*TMEM156*	-	-		-	-	-	-	-	-	-	-	-	1.00

sEVs, small extracellular vesicles; BLV, bovine leukemia virus; WBC, white blood cell; PVL, proviral load; and LDH, lactate dehydrogenase.

## Data Availability

The data presented in this study are available within the article and in the Supplementary Material.

## Supplementary Materials

The following supporting information can be downloaded at: https://www.mdpi.com/article/10.3390/pathogens12101239/s1, Table S1: List of selected 23 mRNAs in milk sEVs from BLV-infected cattle; Table S2: Oligonucleotide primers used for qPCR analysis. Figure S1: Low-magnification TEM images of Figure 1 (box), full WB image for surface-, internal-, and contaminant control-marker proteins CD63, HSP70, and apoA1, respectively, in milk sEVs from control and BLV-infected cattle. Figure S2: Relative expression level of mRNAs in milk sEVs in accordance with BLV copy numbers. References [32,33,34,35,36,37,38,39,40,41,42,43,44,45,46,47,48,49,50,51,52,53,54] are cited in the supplementary materials.
