# Correction: Rahman et al. Characterization of mRNA Signature in Milk Small Extracellular Vesicles from Cattle Infected with Bovine Leukemia Virus. *Pathogens 2023*, *12*, 1239

**DOI:** 10.3390/pathogens14050434

**Published:** 2025-04-30

**Authors:** Md. Matiur Rahman, Hinata Ishikawa, Marika Yamauchi, Shigeo Takashima, Yuji O. Kamatari, Kaori Shimizu, Ayaka Okada, Yasuo Inoshima

**Affiliations:** 1 Laboratory of Food and Environmental Hygiene, Cooperative Department of Veterinary Medicine, Gifu University, Gifu 501-1112, Japan; 2 Department of Medicine, Faculty of Veterinary, Animal and Biomedical Sciences, Sylhet Agricultural University, Sylhet 3100, Bangladesh; 3 Division of Genomics Research, Life Science Research Center, Gifu University, Gifu 501-1112, Japan; 4 Institute for Glyco-Core Research (iGCORE), Gifu University, Gifu 501-1112, Japan; 5 The United Graduate School of Drug Discovery and Medical Information Sciences, Gifu University, Gifu 501-1112, Japan; 6 Division of Instrumental Analysis, Life Science Research Center, Gifu University, Gifu 501-1112, Japan; 7 Education and Research Center for Food Animal Health, Gifu University (GeFAH), Gifu 501-1112, Japan; 8 Joint Graduate School of Veterinary Sciences, Gifu University, Gifu 501-1112, Japan

## 1. Error in Figure

In the original publication [1], there was an error in Figure 1. Specifically, there was a mistake in the transmission electron microcopy images of control cattle (Figure 1a) and the Western blot images of HSP70 and apoA1 (Figure 1c). The corrected Figure 1 is shown below. 

## 2. Figure Legend

Figure S1 is directly related to Figure 1, and as a result, it is also affected by the error. Since the updated Figure 1 required modifications, Figure S1 was revised accordingly to ensure consistency in the presented data. The corrected Figure S1 is shown below.

## 3. Table Footnote

There was an error in the original publication in the footnote of Table 1. A key explanatory note was omitted from the table footnote, which led to incomplete information. The missing details have now been added for clarity. The corrected table footnote is provided below.

+, positive; -, negative; NT, not tested; BLV, bovine leukemia virus; sEVs, small extra cellular vesicles; qPCR, quantitative real-time polymerase chain reaction; ELISA, enzyme-linked immunosorbent assay; PVL, proviral load; WBC, white blood cell; Key of EC, leukosis-key of the European Community (-, normal; ±, suspect, +, lymphocytic) [58]; LPVL, low proviral load; HPVL, high proviral load; and LDH, lactate dehydrogenase. Some of the blood examination data were obtained from our previously published paper [31]. 

## 4. Supplementary Materials Statement

There was an error in the original publication in the Supplementary Materials statement. As Supplementary Figure S1 was updated, the correct statements should be as follows:

**Supplementary Materials:** The following supporting information can be downloaded at: https://www.mdpi.com/article/10.3390/pathogens12101239/s1. Table S1: List of selected 23 mRNAs in milk sEVs from BLV-infected cattle; Table S2: Oligonucleotide primers used for qPCR analysis. Figure S1: Low-magnification TEM images of Figure 1 (box), full WB image for surface-, internal-, and contaminant control-marker proteins CD63, HSP70, and apoA1, respectively, in milk sEVs from control and BLV-infected cattle. Figure S2: Relative expression level of mRNAs in milk sEVs in accordance with BLV copy numbers. References [32–54] are cited in the supplementary materials.

The authors state that the scientific conclusions are unaffected. This correction was approved by the Academic Editor. The original publication has also been updated.

## Figures and Tables

**Figure 1:**
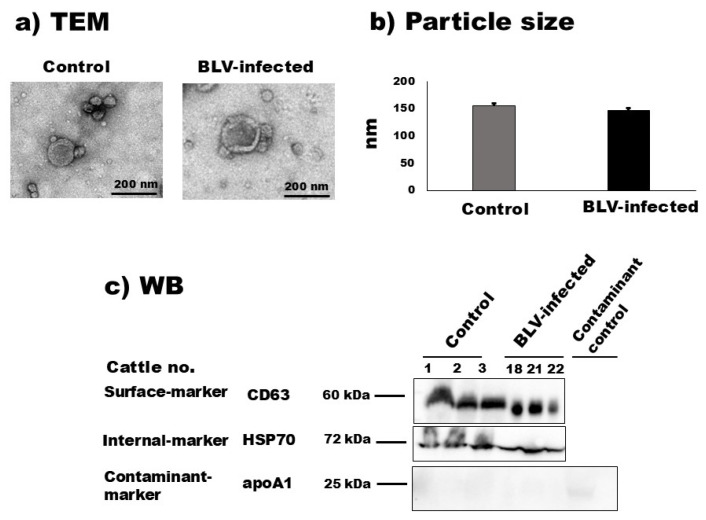
Characterization of milk small extracellular vesicles (sEVs). (**a**) Morphological features of bovine milk sEVs from control cattle and bovine leukemia virus (BLV)-infected cattle were observed by transmission electron microscopy analysis (scale bar indicates 200 nm). (**b**) Nanoparticle tracking analysis determined the size distribution of milk sEVs from control and BLV-infected cattle (average mean peak size below 200 nm in diameter), and there was no significant difference observed between them (*p* > 0.05). (**c**) Bovine milk sEVs surface-, internal-, and contaminant-marker proteins, CD63, HSP70, and apoA1, were detected by Western blot analysis, indicating that bovine milk sEVs were successfully isolated according to the minimal information for studies of extracellular vesicles 2018 guidelines [17]. BLV, bovine leukemia virus; TEM, transmission electron microscopy; and WB, Western blot.

**Figure S1:**
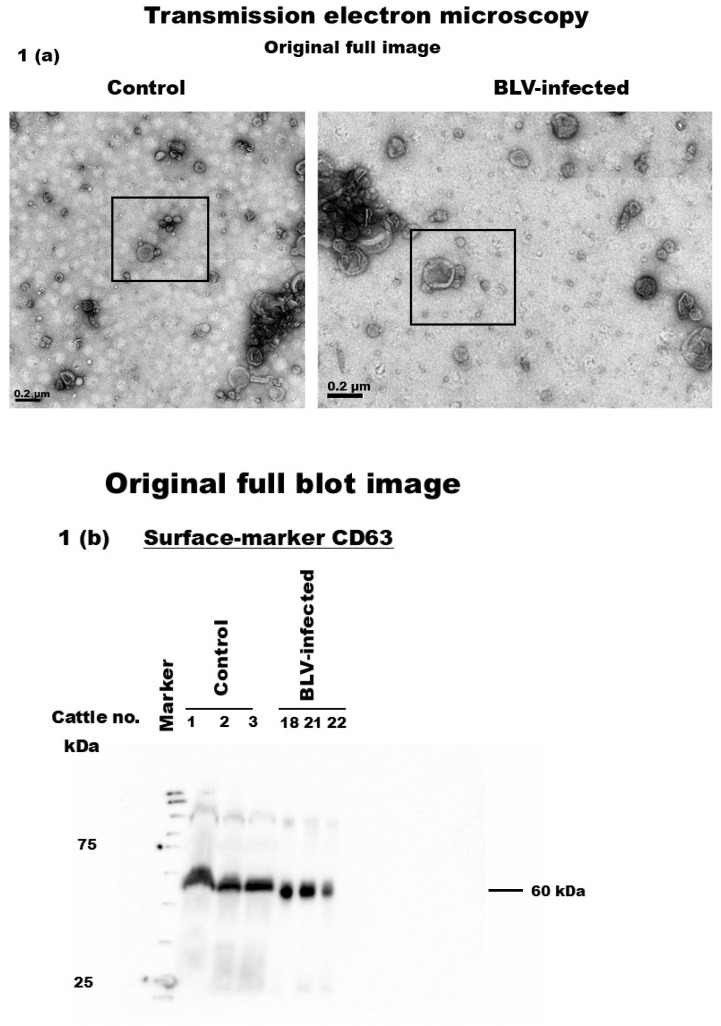

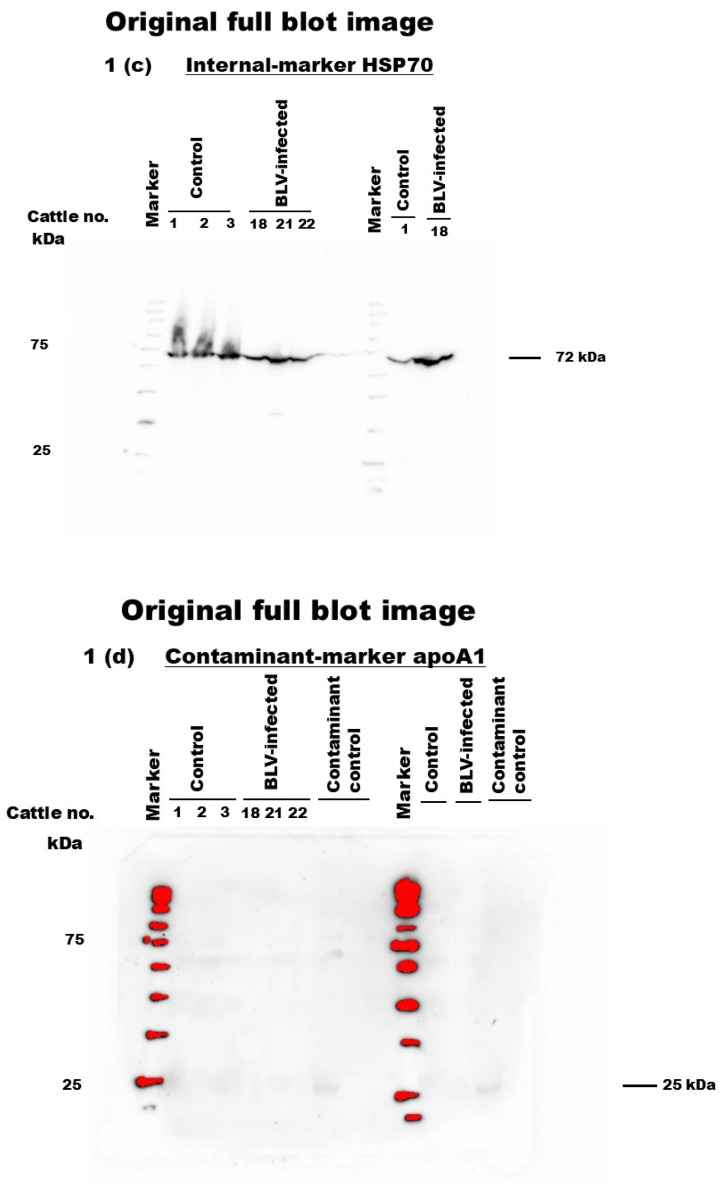
Low-magnification TEM images of Figure 1 (box), full WB image for surface-, internal-, and contaminant control-marker proteins CD63, HSP70, and apoA1, respectively, in milk sEVs from control and BLV-infected cattle.
