# Supplementary material for: Characterization of mRNA Signature in Milk Small Extracellular Vesicles from Cattle Infected with Bovine Leukemia Virus
Source: Pathogens. 2023 Oct 13;12(10):1239. doi: 10.3390/pathogens12101239 (PMC10610248; doi:10.3390/pathogens12101239)
Supplement: Supplementary file 1 [file pathogens-12-01239-s001.zip › pathogens-2598600-supplementary Table S1.pdf]

Supplementary Table S1. Selected 23 mRNA list in milk sEVs from BLV-infected cattle.

Gene name	Gene symbol	Fold change	Functions	Reference
Myoferlin	*MYOF*	12.67	Tumor metastasis	[32]
Transmembrane protein 156	*TMEM156*	10.79	Cancer invasion	[33]
Interleukin 33	*IL33*	6.06	Cancer metastasis	[34]
Ubiquitin conjugating enzyme E2 C	*UBE2C*	5.61	Tumor progression	[35]
Microtubule associated protein 9	*MAP9*	3.50	Cancer progression	[36]
Pyruvate dehydrogenase kinase 3	*PDK3*	3.17	Tumor proliferation	[37]
SPC24 component of NDC80 kinetochore complex	*SPC24*	3.14	Tumorigenesis	[38]
Cyclin B1	*CCNB1*	2.50	Cellular proliferation	[39]
MHC class I JSP.1	*JSP.1*	2.46	Apoptosis	[40]
Transforming growth factor beta receptor 1	*TGFBR1*	1.51	Cellular-metabolism	[41]
Mohawk homeobox	*MKX*	1.50	Tissue differentiation	[42]
Bovine major histocompatibility complex	*BoLA*	1.28	BLV infectivity	[43]
Mitochondrial ribosomal protein L1351	*L13*	1.26	Immune response	[44]
Synaptosome associated protein 47	*SNAP47*	1.14	Cancer invasion	[45]
Succinate dehydrogenase complex flavoprotein subunit A	*SDHA*	1.14	Cellular metabolism	[46]
Cell division cycle 20	*CDC20*	1.13	Cellular proliferation	[47]
Arginine and serine rich protein 1	*RSRP1*	1.08	Cell invasion	[48]
Apoptosis resistant E3 ubiquitin protein ligase 1	*AREL1*	1.01	Anti-apoptosis	[49]
Calbindin 1	*CALB1*	-1.51	Metabolism	[50]
Integrin subunit beta 2	*ITGB2*	-1.39	Cancer proliferation	[51]
Hypoxanthine phosphorribosyl transferase 1	*HPRT*	-1.13	Cell cycle progression	[52]
Stomatin like 2	*STOML2*	-1.03	Metastasis	[53]
WD repeat domain 89	*WDR89*	-1.02	Cell cycle	[54]
