# Supplementary material for: Characterization of mRNA Signature in Milk Small Extracellular Vesicles from Cattle Infected with Bovine Leukemia Virus
Source: Pathogens. 2023 Oct 13;12(10):1239. doi: 10.3390/pathogens12101239 (PMC10610248; doi:10.3390/pathogens12101239)
Supplement: Supplementary file 1 [file pathogens-12-01239-s001.zip › pathogens-2598600-supplementary Table S2.pdf]

Supplementary Table S2. Oligonucleotide primers used for qPCR analysis.

Gene symbol	Primer sequences (5'-3')
AREL1	F: GGACCTCGTTTAAAGCTGCTGTG<br>R: TATAGTCCGGTCCCCTCGGC
BoLA	F: CGGAAGGTTATGCTGAGGTACA<br>R: AGCGTGTCCTTCCCATTCTC
CALB1	F: CGCCCTGAGTTCCCTTCTCC<br>R: AGGTGGGATTCTGCCATCGT
CCNB1	F: TGGGTCGGCCTCTACCTTTGCACTTC<br>R: CGATGTGGCATACTTGTTCTTGATAGTCA
CDC20	F: AGTCTGACCATGAGCCCAGA<br>R: ATGCCTTGGTGGATGAGGC
HPRT	F: TGAGGATTTGGAAAGGGTGT<br>R: GAGCACACAGAGGGCTACAA
IL33	F: AATGCTCAGCAGGCAAAGCC<br>R: TCAGCTGCTCACAGGCAACT
ITGB2	F: CCCAGGATGCACCAACTAC<br>R: TGTGTCACAGCGAATGGAGT
JSP.1	F: CAGGAGGGGCCGGAGTATTG<br>R: TAGCCGAACTGCCAGAACCC
L13	F: CGGACCGTGCGAGGTAT<br>R: CACCATCCGCTTTTTCTTGTC
MAP9	F: CAGTGCATCTGGCAGATTAATGAC<br>R: GCTGTCGACGGAATCCTCC
MKX	F: AATCGCCCCTTTTGCTCCCT<br>R: GTGTCTCAGGCCCAGGTTGT
MYOF	F: TCTATCAAGCCAGGAACCTCATG<br>R: GGTTTTACTTCGATGAAGGAAGGA
PDK3	F: TGAAGCTGACGATTGGAGCA<br>R: CATCTTGTCCTGTTTTGCCTTGT
RSRP1	F: TGGGCAAATGCCTTAGTTCTTTT<br>R: GGCTTTGCTACAGAATTGTTAGAGC
SDHA	F: TTTGATGCAGTGGTGGTAGG<br>R: CAGAGCAGCATTGATTCCTC
SNAP47	F: GAGTCCTCCACCATCAGGGC<br>R: CCGATGGCGTCTTCCAAAGC
SPC24	F: TGCGAGAGATCCTCACCACA<br>R: TTGTGAGCTGAAGGAGGTTGG
STOML2	F: CCCGGCGACGTTACCAGTAT<br>R: GGCAGGGAGGCTAGAACCAG
TGFBR1	F: CAGGTTTACCATTGCTTGTTCA<br>R: TGCCATTGTCTTTATTGTCTGC
TMEM156	F: GTGCAGAAGTGGCAGAGTCAT<br>R: AGGAAGCACGTCCTTGACTG
UBE2C	F: ACGGTGAAGTTCCTCACACC<br>R: AGAACACAGGGAGAGCTGGA
WDR89	F: TCTGTGCTCGAGTCCGTTCC<br>R: AGGTTCAAGTGGTTGGTGCCT
