# Supplementary material for: Characterization of mRNA Signature in Milk Small Extracellular Vesicles from Cattle Infected with Bovine Leukemia Virus
Source: Pathogens. 2023 Oct 13;12(10):1239. doi: 10.3390/pathogens12101239 (PMC10610248; doi:10.3390/pathogens12101239)
Supplement: Supplementary file 1 [file pathogens-12-01239-s001.zip › Supplementary Figure S1 and Figure S2.pdf]

# Transmission electron microscopy

**1 (a)**

## Original full image

**Control** **BLV-infected**

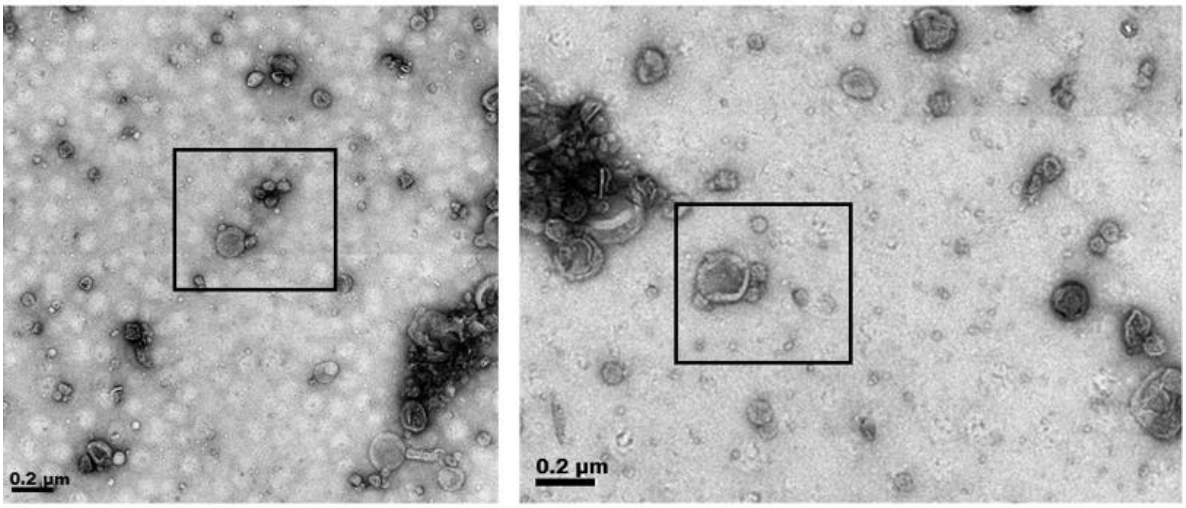


**Figure S1 (a)**

# Original full blot image

**1 (b)** **Surface-marker CD63**

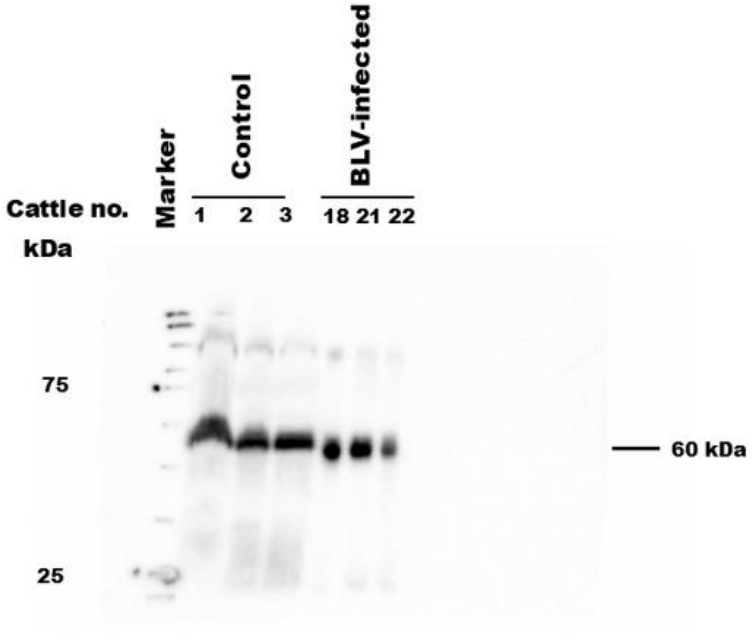


**Figure S1 (b)**

## Original full blot image

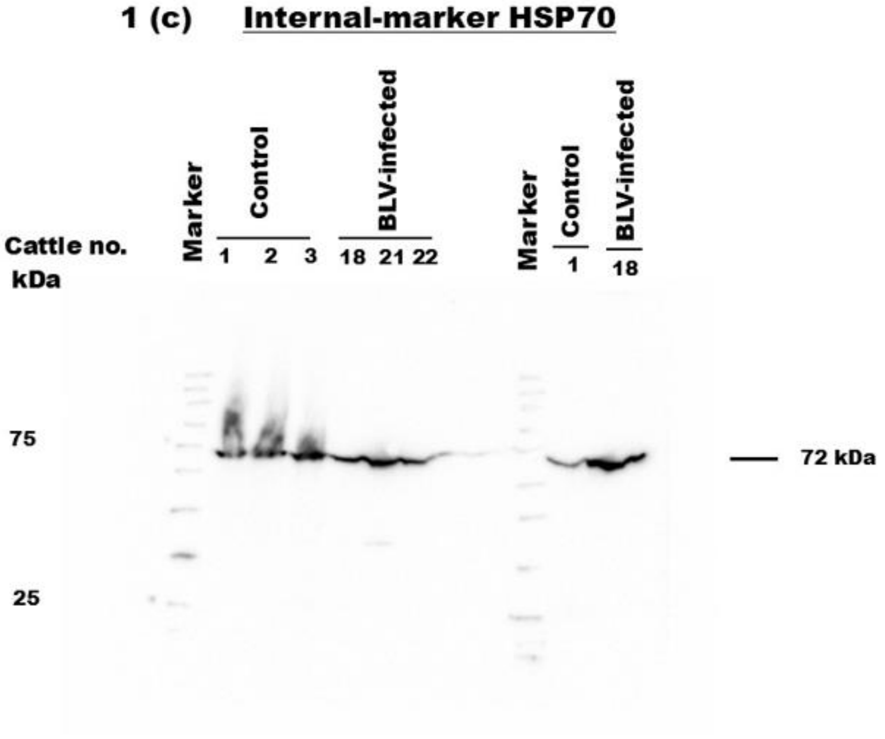


Figure S1 (c)

## Original full blot image

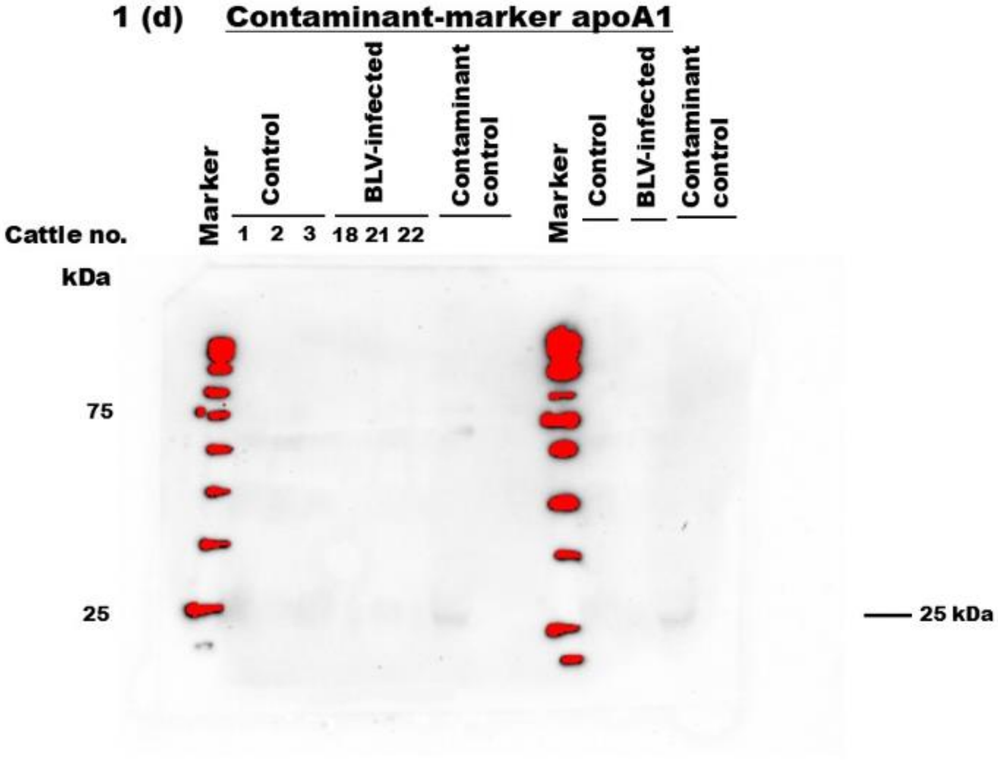


Figure S1 (d)

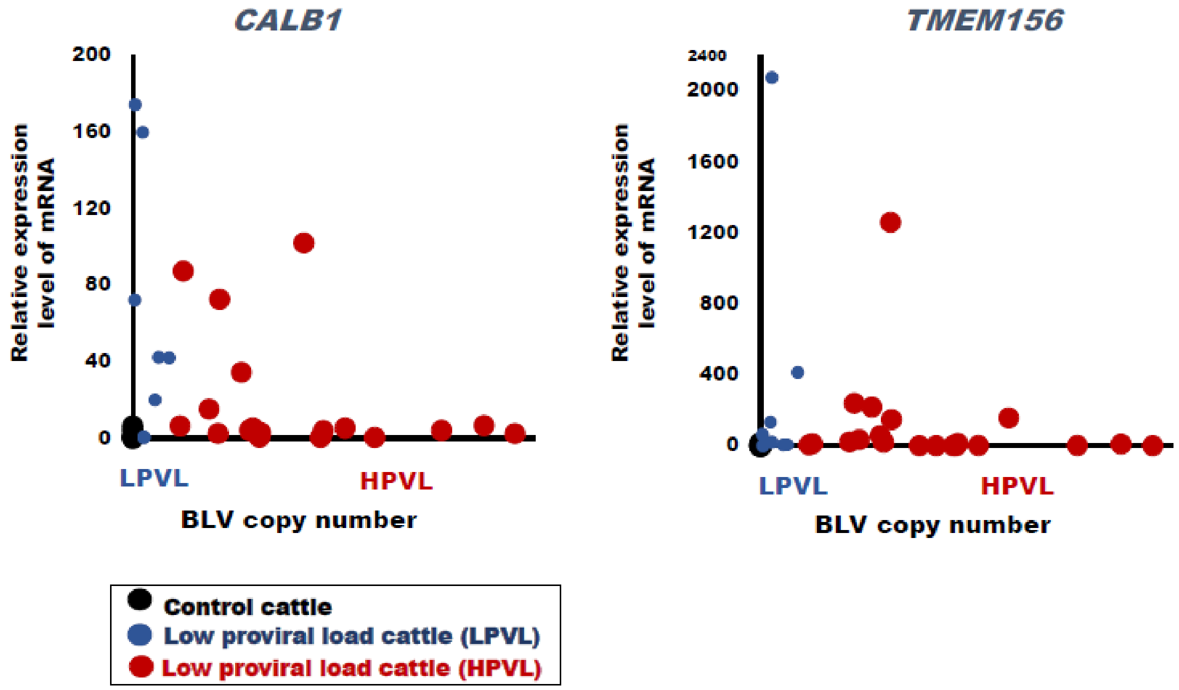


**Supplementary Figure S2**
